# Tuning into Places

**DOI:** 10.1371/journal.pbio.1001042

**Published:** 2011-04-05

**Authors:** Rachel Jones

**Affiliations:** Freelance Science Writer, Welwyn, Hertfordshire, United Kingdom

**Figure pbio-1001042-g001:**
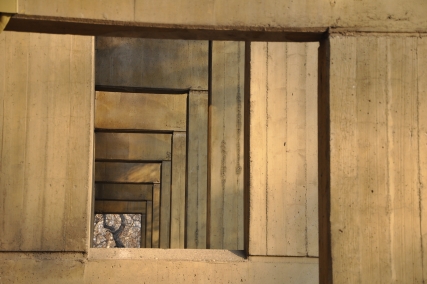
The spatial layout of a scene can be characterized by edges and borders, which tend to have higher spatial frequencies. Image credit: Photograph by Arash Afraz.

[Fig pbio-1001042-g001]In the human brain, the inferior temporal cortex contains areas that seem to respond selectively to particular types of visual stimuli, such as faces or animals. One such region—the parahippocampal place area, or PPA—responds most strongly to images of places instead of faces, but how this higher-order selectivity is achieved remains unclear. In a new study in *PLoS Biology*, Rajimehr et al. identify a specific response property of the PPA that might underlie its selectivity for place images. The PPA, the authors show, responds preferentially to images with high spatial frequencies—that is, with a high number of contrasting elements within a given space on the retina—corresponding to the type of details one might see in natural scenes.

The authors used functional MRI to investigate how images of simple three-dimensional shapes evoked activity in different parts of the visual cortex in humans. Unexpectedly, they found that the PPA responded much more strongly to a cube than a sphere, despite the fact that neither represented a “place” of any kind. The two images were carefully matched for many visual properties, but one property that differed was the presence of the edges and corners in the image of the cube. Images can be characterized by whether they contain mainly low, medium, or high spatial frequencies; the cube image contains more high spatial frequencies than the sphere.

To investigate whether the presence of high spatial frequencies was indeed responsible for the difference in the way the PPA responded to the cube and the sphere, the authors used even simpler visual stimuli—flickering checkerboard patterns—and found that these also caused selective activation of the PPA—but only when they contained high spatial frequencies. Next, the authors used spatial filters to alter standard images of faces and places so that they contained only low, medium, or high spatial frequencies, and tested the ability of these filtered images to activate the PPA.

The results showed that the PPA responded strongly to images containing high spatial frequencies, whether they showed faces or places. Notably, even though faces do not normally activate the PPA, images of faces from which low and medium spatial frequencies had been removed (leaving only high spatial frequencies) activated the PPA as strongly as did place images.

It is worth noting that the PPA is defined not anatomically, but functionally, by comparing responses to natural images of faces and places in the inferior temporal cortex. Using their new data, Rajimehr et al. show that the topography of the PPA localized in this traditional way was mirrored by the area that showed a bias for high spatial frequencies.

Next, the authors carried out similar studies in macaque monkeys. Although the inferior temporal cortex of macaques shares many functional features with that of humans, a place-selective homolog of the PPA has not been discovered in the macaque brain. Rajimehr et al. used the same stimuli that they used in the human studies to show that macaque inferior temporal cortex does contain a place-selective area homologous to the human PPA. This region also showed the same selectivity for high spatial frequencies as the human PPA, revealing an important parallel between the organization of the human and monkey cortex.

Images of places or natural scenes typically contain spatial discontinuities and high spatial frequencies, often in the form of the edges of buildings or tree trunks. Thus it follows that the PPA's preference for high spatial frequencies might contribute to its robust response to place images. The authors point out that further research would benefit from quantitative comparisons between responses to place images and those to images containing different spatial frequency distributions, in addition to the qualitative comparisons carried out in this study. However, such experiments will require researchers to develop a way to quantify the “placeness” of an image. Future research will likely take advantage of the demonstration that macaque brains contain a PPA homolog by carrying out electrophysiological studies that would be impossible in humans. Such studies may help identify the neural mechanisms underlying place selectivity in the human brain and perhaps even shed light on the precursors of place recognition in our primate cousins.

**Rajimehr R, Devaney KJ, Bilenko NY, Young JC, Tootell RBH (2011) The “Parahippocampal Place Area” Responds Preferentially to High Spatial Frequencies in Humans and Monkeys. doi:10.1371/journal.pbio.1000608**

